# Mathematical modeling and computer simulation of the three-dimensional pattern formation of honeycombs

**DOI:** 10.1038/s41598-019-56942-6

**Published:** 2019-12-30

**Authors:** Darae Jeong, Yibao Li, Sangkwon Kim, Yongho Choi, Chaeyoung Lee, Junseok Kim

**Affiliations:** 1 Department of Mathematics, Kangwon National University, Gangwon-do, 24341 Republic of Korea; 2 School of Mathematics and Statistics, Xi’an Jiaotong University, Xi’an, 710049 China; 3 Department of Mathematics, Korea University, Seoul, 02841 Republic of Korea; 4 Department of Mathematics and Big Data, Daegu University, Gyeongsan-si, Gyeongsangbuk-do 38453 Republic of Korea

**Keywords:** Applied mathematics, Computational science

## Abstract

We present a mathematical model, a numerical scheme, and computer simulations of the three-dimensional pattern formation of a honeycomb structure by using the immersed boundary method. In our model, we assume that initially the honeycomb cells have a hollow hemisphere mounted by a hollow circular cylinder shape at their birth and there is force acting upon the entire side of the cell. The net force from the individual cells is a key factor in their transformation from a hollow hemisphere mounted by a hollow circular cylinder shape to a rounded rhombohedral surfaces mounted by a hexagonal cylinder shape. Numerical simulations of the proposed mathematical model equation produce the rounded rhombohedral surfaces mounted by a hexagonal cylinder patterns observed in honeybee colonies.

## Introduction

**Figure 1:**
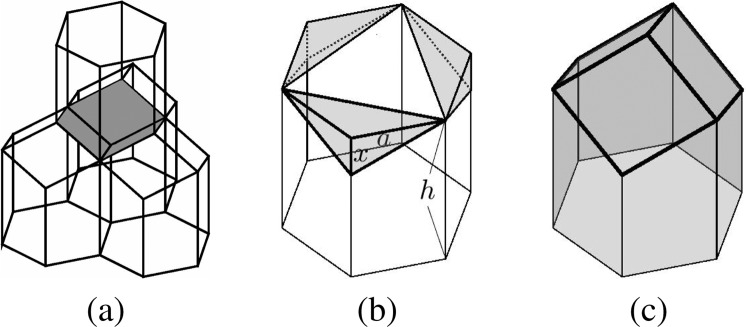
Three-dimensional honeycomb structure. (**a**) Schematic illustration of four unit honeycomb cells. (**b**) Hexagonal cylinder, and (**c**) three-dimensional geometry of a honeycomb cell.

Although it is well known that the shape of the honeycomb cell is hexagonal, the three-dimensional structure of it is less known. Actual honeycombs consist of two layers of congruent cells, each one with a hexagonal opening and is capped by three rhombic faces^1^. Figure 1a represents a schematic illustration of four unit honeycomb cells.

Let us consider a hexagonal cylinder with side $a$ and height $h$, see Fig. 1b, then volume is $V=1.5\sqrt{3}{a}^{2}h$. If we cut the three shaded tetrahedra and put them together on the top of the hexagonal cylinder, then we have a three-dimensional geometry of a honeycomb cell as shown in Fig. 1c. The area of the surface with one open side is $S=3a(2h-x)+3\sqrt{3}a\sqrt{0.25{a}^{2}+{x}^{2}}$, where $x$ is the height of the shaded tetrahedron. With a fixed volume, i.e., $a$ and $h$ are fixed, $S$ has the maximum value at $x=a/(2\sqrt{2})$.

Karihaloo *et al*.^2^ reported that the fresh cells in a natural honeybee comb have a circular shape however quickly transform into the rounded hexagonal structure, while the comb is being built. Bird nests in nature are typically hemispherical in shape. In this study, it is proposed that rounded hexagonal and rhombic pattern formation in the honeybee comb is the result of the net force coming from the forces exerted outward by the cell boundaries. This work extends a previous study of honeycomb formation that only considered two dimensions^3^; we examine the structure from a three-dimensional perspective.

## Simulation Results

**Figure 2:**
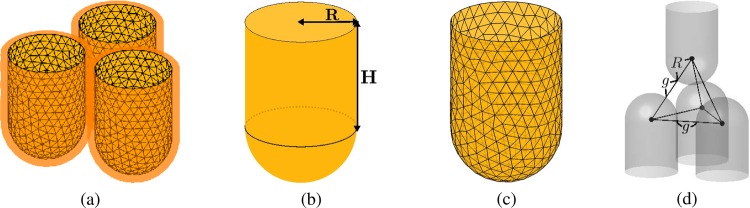
(**a**) Initial array of cells. (**b**) Hollow hemisphere mounted by a hollow cylinder as the initial cell. (**c**) Surface triangulation of the unit cell. (**d**) Gap distance between the cells by $g$.

The proposed method was implemented using C language and the visualization of results was performed using MATLAB software (The MathWorks, Natick, MA, USA). For simulation, we assume the initial array of cells as shown in Fig. 2a, where each cell contacts other cells and has thin soft layer. We consider the initial unit cell as a hollow hemisphere mounted by a hollow cylinder as shown in Fig. 2b. The radius of the hemisphere is $R=1$ with a center $(x,y,z)=(0,0,1)$ and the height of the cylinder is $H=2$. We generate the surface triangulation of the unit cell using DistMesh^4^, which is an open source MATLAB mesh generator, see Fig. 2c.

**Figure 3:**
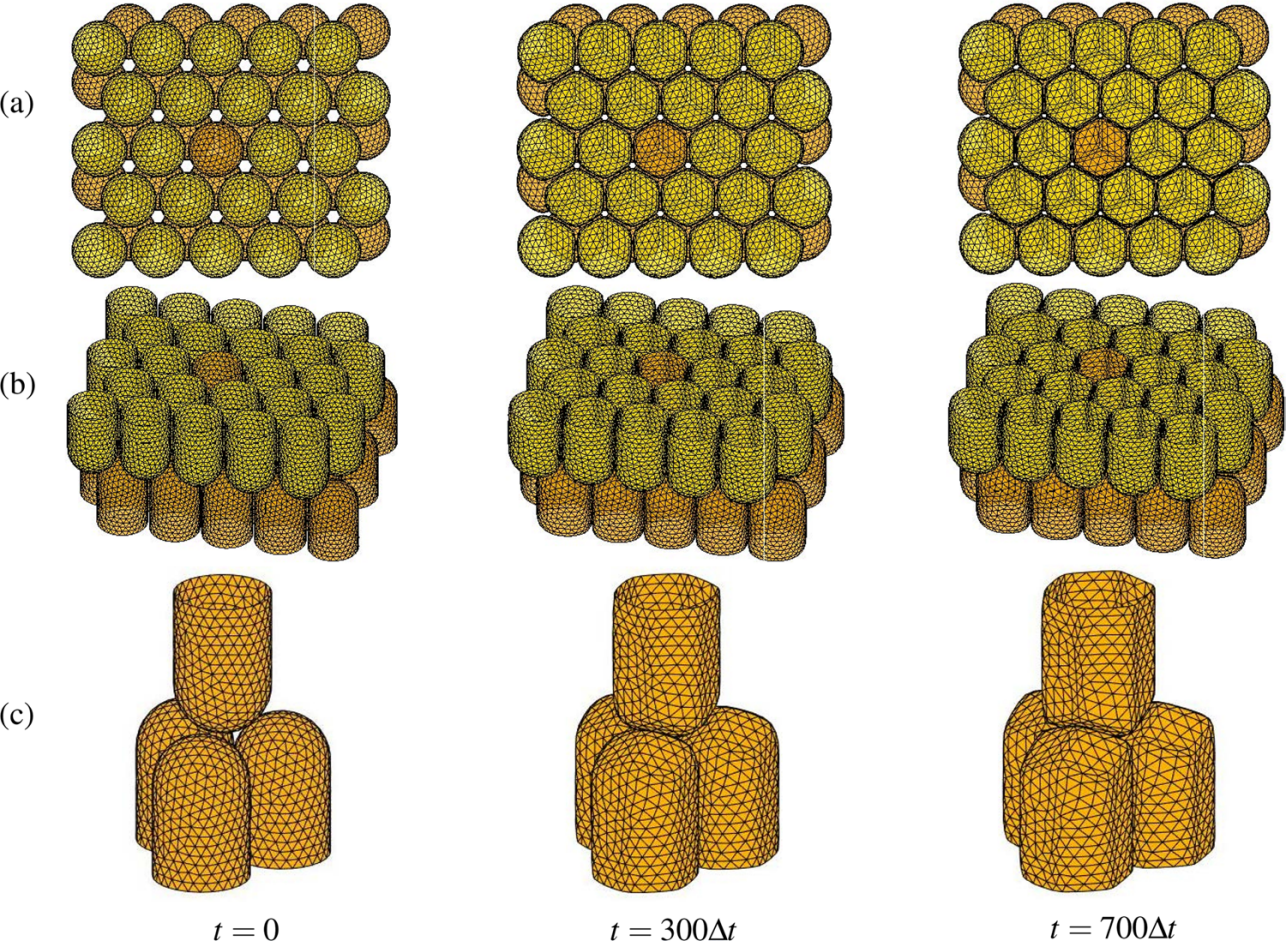
Time evolution at $t=0,300\Delta t$, and $700\Delta t$: (**a**) top view, (**b**) side view, and (**c**) part of (**b**).

We investigate the temporal evolution of $50$ cells by the proposed model. We denote the gap distance between the cells by $g$, see Fig. 2d. Figure 3 shows the snapshots at time $t=0,300\Delta t$, and $700\Delta t$. Here, we use ${N}_{x}=73$, ${N}_{y}=63$, ${N}_{z}=50$, $h=0.2496$, $\Delta t=0.1{h}^{2}$, and $g=0.1$. Figure 3a,b are two different views. To examine the evolution of the cells in detail, we isolate four cells including the highlighted cell from the other cells as shown in Fig. 3c. We can observe the cells transform from a circular cylinder to a rounded hexagonal cylinder and from a hemisphere to a rounded rhombohedral surface.

**Figure 4:**
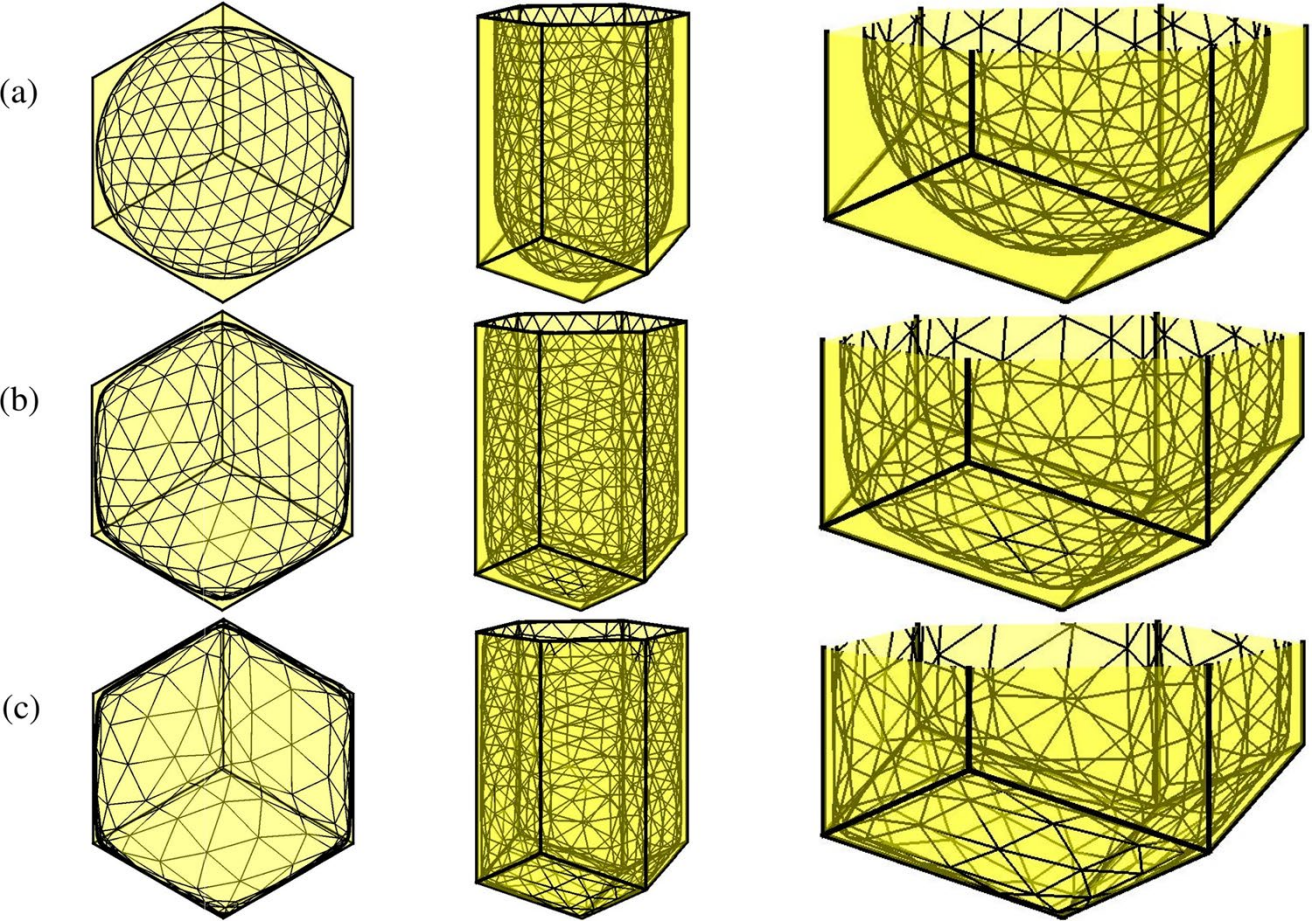
Different views of the snapshots of the highlighted cell with a reference frame at (**a**) $t=0$, (**b**) $t=120\Delta t$, and (**c**) $t=600\Delta t$.

Let us consider the morphology evolution of the highlighted cell in more detail. Figure 4a–c show the different views of the snapshots of the highlighted cell with a best fitted reference frame at $t=0,120\Delta t$, and $600\Delta t$, respectively. The reference frame is the mathematically optimized shape. The result indicates that the highlighted cell is getting fit to the optimized shape, which is observed in nature.

**Figure 5:**
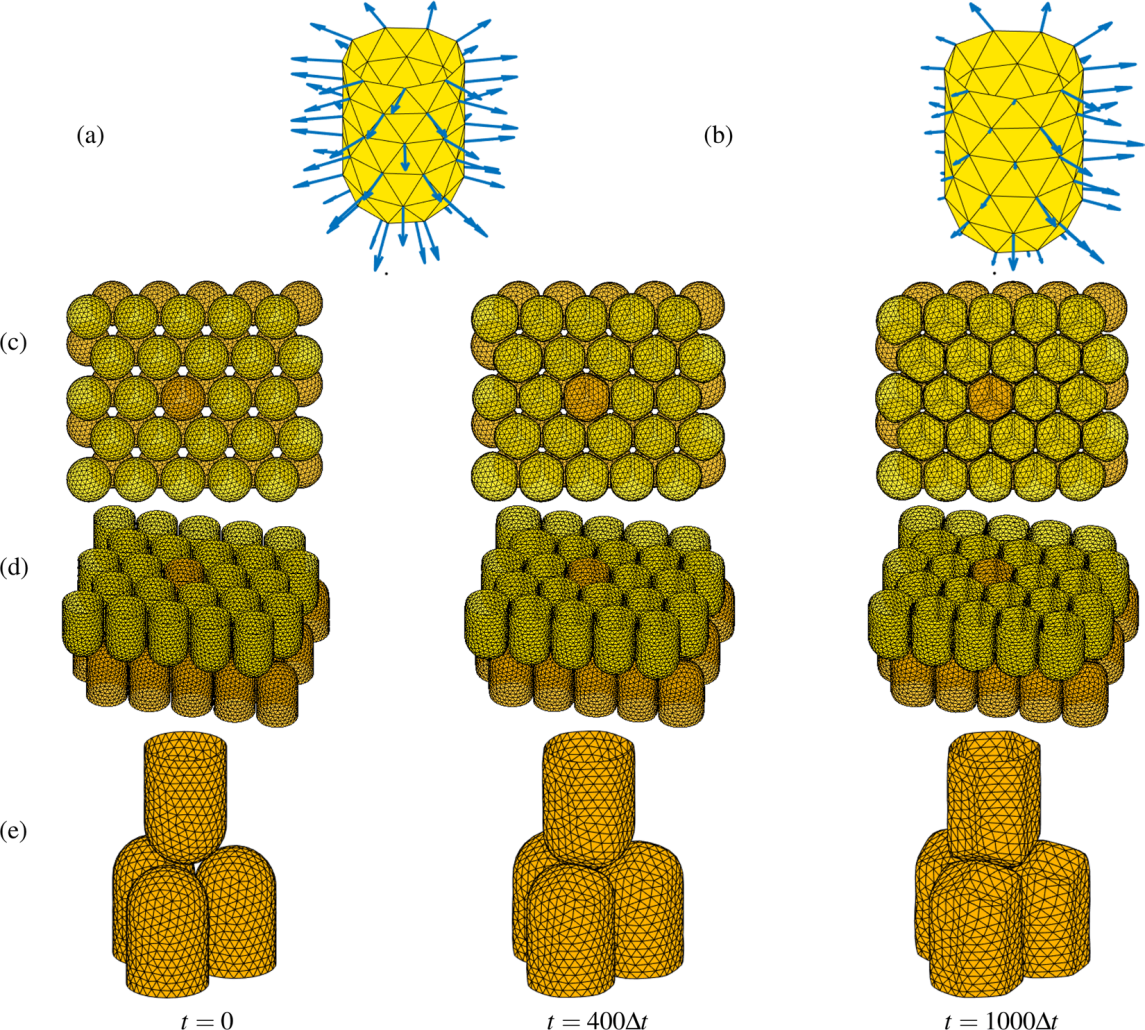
Schematic of boundary force with (**a**) constant value and (**b**) random value. Time evolution of randomly forcing cells at $t=0,400\Delta t$, and $1000\Delta t$: (**c**) top view, (**d**) side view, and (**e**) part of (**d**).

So far, we performed computational tests with uniform boundary force as shown in Fig. 5a. As the final test, we investigate the temporal evolution of $50$ cells with random boundary force as shown in Fig. 5b. Numerical parameters are the same in the previous test except time step, $\Delta t$. Here, we use time step size as $\Delta t={h}^{2}$. The boundary force is randomly given in space and time, i.e., we randomly apply boundary force to 10% of cells. The numerical results are represented in Fig. 5c–e. Figure 5c,d are two different views and Fig. 5e is the four cells including the highlighted cell. Here, we can observe the cells transform from a circular cylinder to a rounded hexagonal cylinder and from a hemisphere to a rounded rhombohedral surface.

## Conclusions

We proposed a mathematical model, a numerical scheme, and computer simulations of the 3D pattern formation of a honeycomb structure using the IBM. In the proposed model, the cells having a hollow hemisphere mounted by a hollow circular cylinder shape at their birth becomes an optimized honeycomb structure by the net force from the outward forces of the individual cells. Computational experiments demonstrated the rounded rhombohedral surfaces mounted by hexagonal cylinder patterns observed in nature. In this paper, we focused on the mathematical modelling for the three-dimensional honeycomb formation and its numerical method. As the future work, it would be interesting to compare the theoretical results with those of experiments.

## Method

In this paper, we propose a mathematical model and perform computational simulations of the three-dimensional honeycomb formation using the immersed boundary method (IBM)^5–7^. The basic mechanism of the three-dimensional model is similar to that of the two-dimensional model^3^ and is as follows: We set the two layers of congruent cells and each cell consists of a hollow hemisphere mounted by a hollow cylinder.

First, we calculate forces acting on the entire surface of the individual cells. Second, we compute the net force from those forces. Third, we move the cell boundary points to new positions according to the net force. We repeat these steps until a specified number of iterations is reached. Now, let us describe the proposed scheme more precisely. We discretize a set of honeycomb cells by a triangular surface mesh $S={\cup }_{p=1}^{{N}_{p}}({V}_{p},{F}_{p}),$ where ${V}_{p}=\{{{\bf{X}}}_{p,q}|1\le q\le {N}_{V}\}$ and ${F}_{p}=\{{T}_{p,q}|1\le q\le {N}_{F}\}$ are the lists of vertices and triangles of a honeycomb cell $p$, respectively.

**Figure 6:**
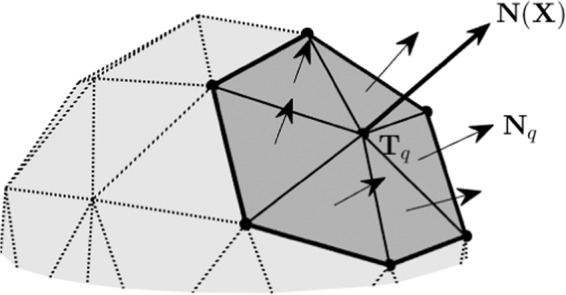
Schematic illustration for the unit normal vector at a vertex.

We use the following evolution equation proposed in^3^:

1 $$\frac{{\rm{d}}{{\bf{X}}}_{p,q}(t)}{{\rm{d}}t}=\alpha {\bf{F}}({{\bf{X}}}_{p,q}(t))\,{\rm{for}}\,p=1,\ldots ,{N}_{p}\,{\rm{and}}\,q=1,\ldots ,{N}_{V},$$

where $\alpha$ is a proportional constant and possess the dimension [time]/[mass]; and ${\bf{F}}$ is the net force resulting from the forces exerted outwardly by the individual cell boundaries such as attaching wax, movement of larva, and storing honey. Let a computational domain embedding the discrete honeycomb cells be partitioned in Cartesian geometry into a uniform mesh with mesh spacing $h$. The center of each grid cell, ${\Omega }_{ijk}$, is located at ${{\bf{x}}}_{ijk}=({x}_{i},{y}_{j},{z}_{k})=((i-0.5)h,(j-0.5)h,(k-0.5)h)$ for $i=1,\cdots ,{N}_{x}$, $j=1,\cdots ,{N}_{y}$, and $k=1,\cdots ,{N}_{z}$. Here, ${N}_{x}$, ${N}_{y}$, and ${N}_{z}$ are the numbers of cells in the $x$-, $y$-, and $z$-directions, respectively. On the Cartesian grid, we applied the zero Dirichlet boundary condition. Let $I({\bf{X}})$ be the set of the index of the triangles containing a vertex ${\bf{X}}$. Then, the unit normal vector at the vertex ${\bf{X}}$ is given by

2 $${\bf{N}}({\bf{X}})=\frac{{\sum }_{q\in I({\bf{X}})}\,{\omega }_{q}{{\bf{N}}}_{q}}{\Vert {\sum }_{q\in I({\bf{X}})}\,{\omega }_{q}{{\bf{N}}}_{q}\Vert },$$

where ${{\bf{N}}}_{q}$ is the unit normal vector of triangle ${T}_{q}$, ${\omega }_{q}=\parallel {{\bf{G}}}_{q}-{\bf{X}}{\parallel }^{-2}$, and ${{\bf{G}}}_{q}$ is the centroid of ${T}_{q}$^8^, see Fig. 6. We define the boundary force per unit surface area ${{\bf{f}}}_{p}^{n}=\sigma {\bf{N}}({{\bf{X}}}_{p}^{n}),$ where $\sigma$ is a magnitude constant.

To compute the net force density, we spread the boundary force over the nearby lattice points:

$${{\bf{F}}}_{ijk}^{n}=\mathop{\sum }\limits_{p=1}^{{N}_{V}}\,{{\bf{f}}}_{p}^{n}{\delta }_{h}^{3}({{\bf{x}}}_{ijk}-{{\bf{X}}}_{p}^{n})\Delta {{\mathscr{A}}}_{p}^{n},$$

where ${\delta }_{h}^{3}(x,y,z)={h}^{-3}\phi (x/h)\phi (y/h)\phi (z/h)$ is a smoothed Dirac delta function^9^:

$$\phi (r)=\{\begin{array}{ll}(3-2|r|+\sqrt{1+4|r|-4{r}^{2}})/8 & {\rm{if}}\,|r| < 1,\\ (5-2|r|+\sqrt{9-4|r|+4{(2-|r|)}^{2}})/8 & {\rm{if}}\,1\le |r|\le 2,\\ 0 & {\rm{otherwise}}\end{array}$$

and $\Delta {{\mathscr{A}}}_{p}^{n}=\sum _{q\in I({\bf{X}})}\,|{T}_{q}|/3$ is a surface element. Here, $|{T}_{q}|$ is the area of the triangle ${T}_{q}$. We set $\Delta {{\mathscr{A}}}_{p}^{n}=\sum _{q\in I({\bf{X}})}\,2|{T}_{q}|/3$ at the points ${{\bf{X}}}_{p}^{n}$ on the boundary points on the top and bottom rims of the cells to compensate the size of surface area because we only have half surface area at the open boundary position. For $p=1,\ldots ,{N}_{V}$, we move the cell boundary points to new positions according to the net force:

3 $${{\bf{X}}}_{p}^{n+1}={{\bf{X}}}_{p}^{n}+\Delta t\mathop{\sum }\limits_{i=1}^{{N}_{x}}\,\mathop{\sum }\limits_{j=1}^{{N}_{y}}\,\mathop{\sum }\limits_{k=1}^{{N}_{z}}\,\alpha {{\bf{F}}}_{ijk}^{n}{\delta }_{h}^{3}({{\bf{x}}}_{ijk}-{{\bf{X}}}_{p}^{n}){h}^{3}.$$

For stability, we move horizontally the points on the top and bottom rims of the cells. This completes the one time step.

## Data Availability

The data that support the findings of this study are available from the corresponding author upon reasonable request.
